# Oleocanthal, an Antioxidant Phenolic Compound in Extra Virgin Olive Oil (EVOO): A Comprehensive Systematic Review of Its Potential in Inflammation and Cancer

**DOI:** 10.3390/antiox12122112

**Published:** 2023-12-14

**Authors:** María González-Rodríguez, Djedjiga Ait Edjoudi, Alfonso Cordero-Barreal, Mariam Farrag, María Varela-García, Carlos Torrijos-Pulpón, Clara Ruiz-Fernández, Maurizio Capuozzo, Alessandro Ottaiano, Francisca Lago, Jesús Pino, Yousof Farrag, Oreste Gualillo

**Affiliations:** 1 SERGAS (Servizo Galego de Saude) and IDIS (Instituto de Investigación Sanitaria de Santiago), NEIRID Lab (Neuroendocrine Interactions in Rheumatology and Inflammatory Diseases), Research Laboratory 9, Santiago University Clinical Hospital, 15706 Santiago de Compostela, Spain; maria.gonzalez3112@gmail.com (M.G.-R.); djidji.aiteldjoudi@gmail.com (D.A.E.); sitoalcorba@gmail.com (A.C.-B.); mariam.r.farrag@gmail.com (M.F.); maria.varela.garcia@sergas.es (M.V.-G.); carlostorrijos00@gmail.com (C.T.-P.); clararf94@gmail.com (C.R.-F.); oreste.gualillo@sergas.es (O.G.); 2 International PhD School of the University of Santiago de Compostela (EDIUS), Doctoral Program in Drug Research and Development, 15782 Santiago de Compostela, Spain; 3 National Health Service, Local Health Authority ASL 3 Napoli Sud, Department of Pharmacy, 80056 Naples, Italy; m.capuozzo@aslnapoli3sud.it; 4 Division of Abdominal Oncology, Istituto Nazionale Tumori IRCCS Fondazione Pascale-IRCCS di Napoli, Ercolano, 80131 Naples, Italy; a.ottaiano@istitutotumori.na.it; 5 Molecular and Cellular Cardiology Group, SERGAS (Servizo Galego de Saude) and IDIS (Instituto de Investigación Sanitaria de Santiago), Research Laboratory 7, Santiago University Clinical Hospital, 15706 Santiago de Compostela, Spain; francisca.lago.paz@sergas.es

**Keywords:** oleocanthal, cancer, inflammation, polyphenolic compounds, extra virgin olive oil (EVOO)

## Abstract

Background: The Mediterranean diet is linked to various health benefits, especially the consumption of olive oil as a key component. Multiple studies highlight its advantages, particularly due to its fatty acid composition and additional components like phenolic compounds. A significant antioxidant compound, oleocanthal, known for its antioxidant properties, has gained attention in the pharmaceutical industry for its anti-inflammatory and antiproliferative effects. It shows promise in addressing cardiovascular diseases, metabolic syndrome, and neuroprotection. This systematic review aims to evaluate the existing literature on oleocanthal, examining its role in biological processes and potential impact on conditions like inflammation and cancer. Methods: We performed several searches in PubMed (MEDLINE), Web of Science (WOS), and Cochrane based on the terms “Oleocanthal”, “Cancer”, and “Inflammation”. The inclusion criteria were as follows: studies whose main topics were oleocanthal and cancer or inflammation. On the other hand, the exclusion criteria were studies that were not focused on oleocanthal, reviews, or editorial material. Given that these findings are explanatory rather than derived from clinical trials, we refrained from employing methods to assess potential bias. This systematic review did not receive any external funding. Results: We found 174 records from these searches, where we discarded reviews and editorial material, duplicated articles, and 1 retracted article. Finally, we had 53 reports assessed for eligibility that were included in this review. Discussion: OC exhibits promising therapeutic potential against both inflammation and cancer. We addressed its ability to target inflammatory genes and pathways, offering potential treatments for conditions like rheumatic diseases by regulating pathways such as NF-kB and MAPK. Additionally, OC’s anticancer properties, particularly its notable inhibition of c-Met signaling across various cancers, highlight its efficacy, showcasing promise as a potential treatment.

## 1. Introduction

Numerous studies have highlighted the advantages of the Mediterranean diet [1]. This diet, renowned for its key lipid component, extra virgin olive oil, has been extensively documented for its beneficial effects in mitigating inflammatory diseases [2], atherosclerosis [3], cancer [4], and neurodegenerative diseases [5].

Multiple phenolic components found in olive oil have undergone extensive research to explore their pharmacological potential [6]. Within extra virgin olive oil (EVOO), an unsaponifiable fraction comprises these phenolic compounds and tocopherol. These constituents function as hydrophilic antioxidants, and they are exclusively found in virgin oil due to their depletion during the refining process [7].

Notably, recent studies have shown that extra virgin olive oil with elevated levels of oleocanthal and oleacein (phenolic compounds), in comparison to standard olive oil, not only enhances the management of obesity and prediabetes but also contributes to an improved inflammatory and oxidative profile [8].

It is worth noting that numerous studies have demonstrated that following a Mediterranean diet is linked to a decreased risk of various cancer types and lower cancer mortality rates [9,10]. The study led by van den Brandt et al. determined that adhering to a Mediterranean dietary pattern could lower the likelihood of developing cancers, specifically lung, postmenopausal breast, esophagus (in men with squamous cell carcinoma), and stomach cancers [10]. A different investigation revealed that individuals who attained higher MEDLIFE scores, signifying stronger adherence to the Mediterranean lifestyle, experienced a 28% reduced risk of cancer-related mortality when contrasted with those who achieved lower MEDLIFE scores [10].

**Figure 1:**
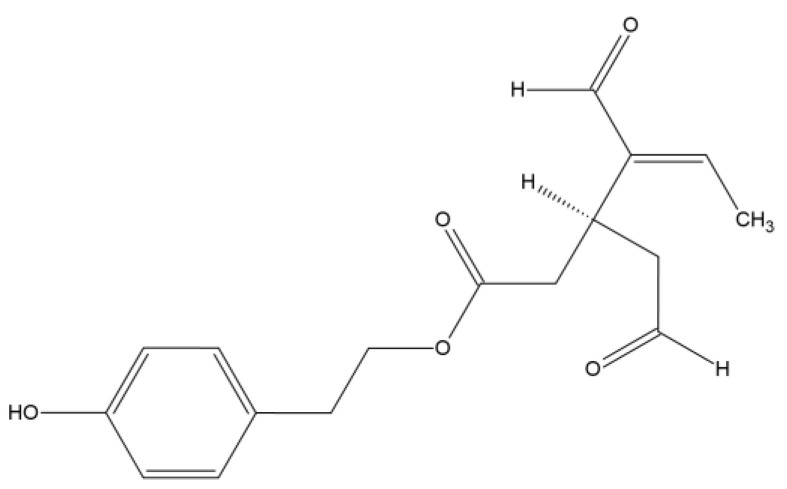
Structure of oleocanthal.

Oleocanthal (OC), a natural phenolic compound found in extra virgin olive oil, was chemically determined in 1993, as the 2-(p-hydroxyphenyl)ethyl ester of (3S)-4-formyl-3-(2-oxoethyl)hex-4-enoic acid (Figure 1) [11]. This compound is garnering increasing attention for its potential role in cancer treatment and inflammation. Extensive research indicates that oleocanthal exhibits anticancer properties, such as inhibiting the growth and proliferation of cancer cells, triggering apoptosis, and reducing angiogenesis [12,13,14]. Additionally, it holds promise for mitigating the adverse effects of radiation and chemotherapy, thereby enhancing their efficacy. These effects are primarily attributed to oleocanthal’s interference with multiple signaling pathways and molecular targets associated with cancer initiation and progression.

As far as we know, the significant presence of oleocanthal in sources other than olive oil has not been extensively reported. The concentration of oleocanthal in extra virgin olive oil can vary significantly, ranging from as little as 0.2 mg/kg to as high as 498 mg/kg. This variability in its content is attributed to factors such as olive cultivars, growing conditions of the olives, agricultural techniques, olive maturity, and processing methods from olives to oil, as well as storage and heating [15].

Geographically, differences exist in the amount of oleocanthal. Italian extra virgin olive oil contains some of the highest concentrations of oleocanthal (up to 191.8 ± 2.7 mg/kg), while EVOOs from the USA have lower amounts (22.6 ± 0.6 mg/kg). Various olive cultivars have shown diverse quantities of oleocanthal, exhibiting almost a tenfold difference between varieties (for example, Coratina has 78.2 ± 0.5 mg/kg and Taggiasca has 8.3 ± 4.0 mg/kg). Additionally, it has been reported that high irrigation of trees decreases the amount of oleocanthal, as well as three-phase centrifugation compared to two-phase [15]. Due to all of these variables, we advocate for the use of oleocanthal as a nutraceutical, defending its consumption in a concentrated and regulated dose that can potentially prevent or treat certain diseases.

The primary objective of this systematic review is to present a comprehensive consolidation of recent discoveries regarding OC, emphasizing its dual properties as an anticancer and anti-inflammatory agent, along with its potential therapeutic role. The review aims to meticulously analyze the current literature to showcase the diverse range of anticancer effects exhibited by OC, including its ability to inhibit cancer cell growth, induce apoptosis, and reduce angiogenesis. Simultaneously, the anti-inflammatory properties of OC will be explored, shedding light on its role in mitigating inflammatory processes.

## 2. Materials and Methods

This systematic review followed the guidelines outlined in the PRISMA statement [16]. We used 3 search engines to conduct this review: PubMed, Web of Science, and the Cochrane Library.

We performed a search on PubMed (MEDLINE) based on the terms oleocanthal, inflammation and cancer. We conducted a search using various combinations of terms: ((Oleocanthal) AND (Inflammation)) OR ((Oleocanthal) AND (Cancer)). The Medical Subject Headings were not included because “oleocanthal” is classified as a Supplementary Concept.

In addition, we performed a search in Web of Science (WOS) using the following forms as topics: ((TS = (oleocanthal)) AND TS = (inflammation)) OR (TS = (cancer) AND TS = (oleocanthal)). To find a topic, the search included the title, abstract, author keywords, and keywords plus. Filtering was performed using the following: Refined By: Document Types: Article; NOT Document Types: Review Article.

The same searches were performed in Cochrane Library, performing a search for “oleocanthal” and “inflammation” and another for “oleocanthal” and “cancer”.

Because we found a limited number of studies, we refrained from filtering the results by the publication year. All of these sources were searched for in October 2023.

The inclusion criteria included studies specifically addressing oleocanthal in the context of cancer or inflammation as their primary focus. Conversely, the exclusion criteria were applied to studies that did not center around oleocanthal, as well as to reviews or editorial materials. The execution of this protocol adhered to the PRISMA checklist and was registered in PROSPERO.

## 3. Results

In PubMed, our search for (Oleocanthal) AND (Inflammation) yielded 42 results spanning from 2010 to 2023. Additionally, a search for (Oleocanthal) AND (Cancer) produced 69 results, covering the period from 2007 to 2023.

In Web of Science (WOS), employing the search query ((TS = (oleocanthal)) AND TS = (inflammation)) OR (TS = (cancer) AND TS = (oleocanthal)), we obtained a total of 113 results. Out of these, 108 were articles, and after excluding 33 review articles, we were left with 77 relevant articles. These articles spanned from 2008 to 2023.

In the case of Cochrane, we performed two distinct searches. The first, using “Oleocanthal” and “Inflammation” as search terms, yielded three results, all published between 2020 and 2023. The second search, with “Oleocanthal” and “Cancer” as keywords, produced four results, and all of which were from the years 2020 to 2023.

**Figure 2:**
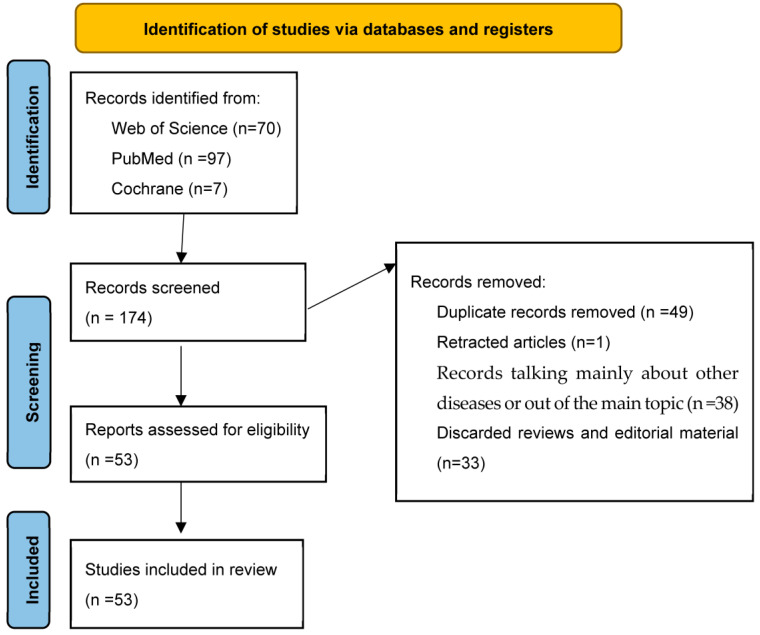
PRISMA 2020 flow chart. This figure summarizes the various stages of this systematic review. The data were collected from September to October 2023 and reviewed in November 2023.

To sum up, we found 174 records from these searches, where we discarded reviews and editorial material, duplicated articles, and 1 retracted article. Finally, we had 53 reports assessed for eligibility that were included in this review (Figure 2).

## 4. Oleocanthal and Inflammation

Oleocanthal has the property of causing a throat tingling sensation when ingested, similarly to ibuprofen. This shared characteristic prompted researchers to question whether this compound could also exhibit anti-inflammatory properties, akin to those of ibuprofen, a non-steroidal anti-inflammatory drug (NSAID). The data collected to date (Table 1) have unequivocally shown that OC effectively suppresses the activity of cyclooxygenase-1 and -2, which are the key enzymes responsible for the production of pro-inflammatory mediators known as prostaglandins, in a dose-dependent manner [17]. In fact, OC is acknowledged as being a naturally occurring NSAID [18,19].

It should be noted that, at equal concentrations, OC was able to inhibit the enzymatic activity of cyclooxigenease-2 (COX-1) and cyclooxigenease-2 (COX-2) with greater potency compared to ibuprofen, making it a promising anti-inflammatory treatment with potential for use as an anticancer drug. It is important to note that COX-2 is an important target for cancer. It is described that is involved in the development of human and animal cancers, being upregulated in this regard [17].

The inflammatory processes within adipose tissue are a pivotal contributor to the onset of numerous chronic conditions linked to obesity. OC emerged as a potent regulator, distinctly suppressing the expression of genes associated with adipocyte inflammation, angiogenesis factors (Vascular Endothelial Growth Factor/Kinase Insert Domain Receptor (VEGF/KDR), Matrix Metalloproteinase-2 (MMP-2)), and oxidative stress markers (Nicotinamide Adenine Dinucleotide Phosphate (NADPH) oxidase), as well as bolstering the activity of antioxidant enzymes like SOD (Superoxide Dismutase) and GPX (Glutathione Peroxidase) [20]. Angiogenesis is triggered and sustained by inflammation, where they mutually amplify each other [21]. Inflammation has been described as an inducer of angiogenesis through the elevation of factors like VEGF due to hypoxia, a process that, as previously mentioned, has the potential to be suppressed by OC [22]. The equilibrium between angiogenic and angiostatic factors dictates the presence and rate of blood vessel growth in tissue, favoring angiogenesis during inflammation. The formation of new blood vessels can facilitate the infiltration of inflammatory cells into affected tissue, perpetuating the progression of disease [21]. In the context of cancer, the inflammatory response within the tumor microenvironment stimulates angiogenesis, conferring several advantages to the tumor, like cellular proliferation, metabolic reprogramming, invasion, and metastasis [23].

Furthermore, OC displayed its remarkable impact by curtailing the chemoattraction and infiltration of leukocytes (Monocyte chemoattractant protein-1 (MCP-1); C-X-C Motif Chemokine Ligand 10 (CXCL-10), Macrophage Colony-Stimulating Factor (MCS-F)) and enhancing the expression of PPARγ (Peroxisome Proliferator-Activated Receptor gamma), an influential mediator of anti-inflammatory and metabolic responses [20]. Moreover, given that platelet activation plays a crucial role in the inflammatory processes linked to atherosclerotic cardiovascular disease, the anti-platelet capability of OC was also studied. A randomized trial showed that OC can affect platelet aggregation responses in healthy adult males, acting as an anti-platelet drug [24]. 

It is important to consider that the antioxidant properties of oleocanthal have also been well documented [25]. In a randomized study that included patients with prediabetes and obesity, two groups were administered different oils: one group consumed oil enriched with oleocanthal and oleacein, while the other group consumed standard oil. The outcome of this study demonstrated that the group consuming the oleocanthal-enriched oil experienced improvements in their inflammatory profile and oxidative condition, along with a reduction in interferon γ levels (IFNγ) [8].

**Figure 3:**
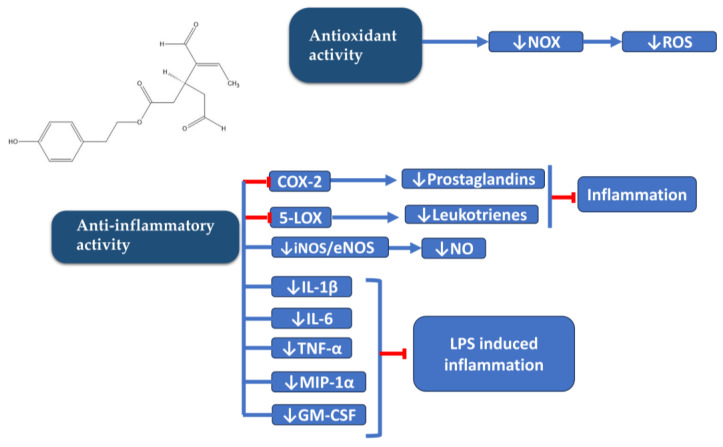
Schematic diagram of anti-inflammatory and antioxidant properties of OC. 5-LOX (5-lipoxygenase); COX 1/2 (Cyclooxygenase 1/2); eNOS (Endothelial Nitric Oxide Synthase); GFAP (Glial Fibrillary Acidic Protein); GM-CSF (Granulocyte-Macrophage Colony-Stimulating Factor); iNOS (Inducible Nitric Oxide Synthase); IL-1β (Interleukin-1β); IL-6 (Interleukin-6); LPS (Lipopolysaccharide); MIP-1α (Macrophage Inflammatory Protein-1α); NO (Nitric Oxide); NOX (Nicotinamide Adenine Dinucleotide Phosphate Oxidase); ROS (Reactive Oxygen Species); TNF-α (Tumor Necrosis Factor-α).

**Figure 4:**
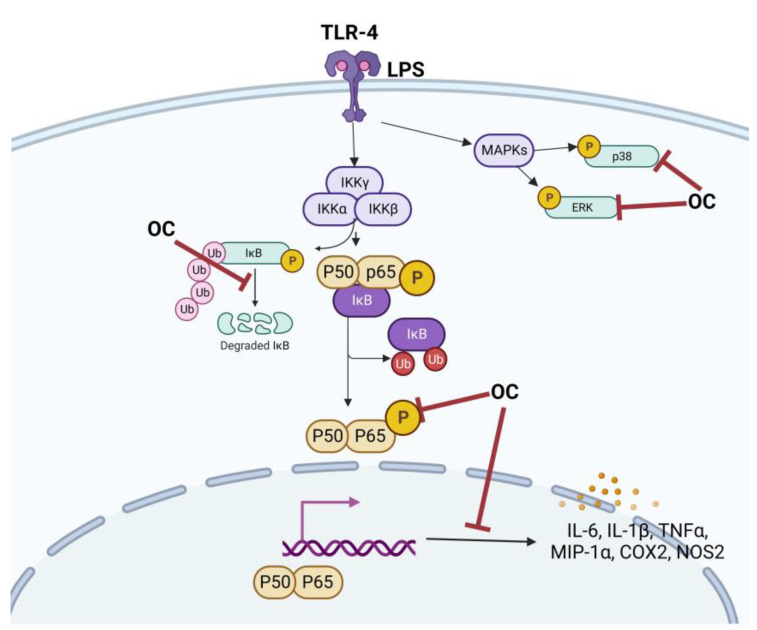
Schematic diagram of anti-inflammatory properties of OC in LPS-induced inflammation. COX-2 (Cyclooxygenase-2); ERK (Extracellular Signal-Regulated Kinase); IL-6 (Interleukin-6); LPS (Lipopolysaccharide); MAPKs (Mitogen-Activated Protein Kinases); MIP-1α (Macrophage Inflammatory Protein-1 alpha); NOS2 (Nitric Oxide Synthase 2); OC (Oleocanthal); p38 (p38 Mitogen-Activated Protein Kinase); TLR4 (Toll-like receptor 4); TNF-α (Tumor Necrosis Factor-alpha); Ub (Ubiquitin).

Scotece et al. also studied the role of OC in multiple myeloma, a neoplastic condition characterized by the clonal proliferation of plasma cells in bone marrow, resulting in bone marrow failure and bone destruction. Within this disease context, OC demonstrated the ability to inhibit the inflammatory protein macrophage inflammatory protein 1 alpha (MIP-1α) [26]. Elevated levels of this protein have been linked to more severe bone disease and reduced survival rates. Notably, OC exhibited the capacity to inhibit this protein not only in myeloma cells but also in chondrocytes and macrophages [19,27]. Scotece et al., in their study of OC in human OA chondrocytes, described its ability to inhibit certain cellular pathways triggered by inflammation, and this led to reduced expression of inflammatory genes, such as Interleukin-6 (IL-6), Interleukin-8 (IL-8), COX-2, Nitric Oxide Synthase-2 (NOS2), MIP-1α, Tumor Necrosis Factor-alpha (TNF-α), and Lipocalin-2 (LCN2), and catabolic genes, such as Matrix Metalloproteinase-13 (MMP13), A Disintegrin, and Metalloproteinase with Thrombospondin Motifs-5 (ADAMTS-5) (Figure 3 and Figure 4) [19]. OC was shown to reduce the phosphorylation of extracellular signal-regulated kinase 1/2 (ERK1/2) and p38 mitogen-activated protein kinases (p-38) caused by LPS in human OA primary chondrocytes (Figure 4). Additionally, OC enhances the expression of IkB, which results in the retention of NF-kB (Nuclear factor kappa-light-chain-enhancer of activated B cells) in the cytosol and a reduction in the nuclear presence of NF-kB p65 triggered by LPS [19]. Regarding rheumatic diseases, a collagen-induced arthritis mouse model exhibited notable improvements in MMP-3 (Matrix Metalloproteinase-3), IL-17 (Interleukin-17), TNF-α, IL-1β (Interleukin-1 beta), IFN-γ, and IL-6 levels when mice were fed with an oleocanthal-rich diet. These levels were significantly reduced in comparison to mice fed a non-oleocanthal-rich diet [28]. Consistent with these findings, Rosillo et al. reported a significant reduction in IL-1β-induced TNF-α and IL-6 release in human synovial SW982 cells when exposed to a polyphenolic extract derived from extra virgin olive oil, primarily composed of oleocanthal and oleacein [29].

With regard to murine peritoneal macrophages stimulated with LPS, antioxidant and anti-inflammatory effects of oleocanthal have been described. These effects have been described to be related with the inhibition of ROS production, MAPKs, and the inflammasome cascade signaling pathway (Figure 4) [30].

In the pursuit of novel strategies to enhance the effectiveness of OC, Montoya and colleagues introduced a methylated derivative of OC, known as (-)-Methyl-oleocanthal (metOC) [31]. This compound has also demonstrated antioxidant and anti-inflammatory properties when tested on murine macrophages stimulated by LPS. Montoya et al. successfully highlighted metOC’s ability to inhibit pro-inflammatory enzymes and cytokines and modulate oxidative products, including nitric oxide (NO) levels and intracellular reactive oxygen species (ROS) [31].

OC has demonstrated a range of beneficial effects on hepatic cells. In vitro investigations have unveiled that OC also effectively reduces cell proliferation and limits the production of the extracellular matrix in LX2 cells, suggesting its antifibrotic properties. Moreover, within HepG2 cells, OC serves to dampen the expression of proinflammatory genes while concurrently boosting the expression of anti-inflammatory genes, underscoring its potential as an anti-inflammatory agent [32]. Furthermore, OC also plays a role in diminishing oxidative stress levels within liver cells, while orchestrating changes in the expression of microRNAs associated with liver fibrosis. In summary, OC shows antifibrotic and anti-inflammatory influences in the liver by ameliorating oxidative stress, mitigating inflammation, and fine-tuning miRNAs connected with liver fibrosis [32].

**Table 1:** Main findings in studies concerning OC and inflammatory diseases.

Condition/Disease	Outcome	Ref.
Osteoarthritis	Inhibition of inflammatory and catabolic mediators in chondrocytes and macrophages: ↓MIP-1α, IL-6, IL-8, COX-2, NOS-2, TNF-α, LCN2, MMP13, and ADAMTS-5	[19,27]
Healthy men	Anti-platelet effects	[24]
Murine peritoneal macrophages	Inhibition of ROS production; inhibition of MAPK pathway; inhibition of the inflammasome cascade signaling pathway; inhibition of IL-1β, IL-6, IL-17, INF-γ, and TNF-α	[30]
Simpson–Golabi–Behmel syndrome adipocytes	↓NFκβ pathway activation ↓VEGF/KDR, MMP-2, NADPH oxidase, SOD, GPX, MCP-1, CXCL-10, MCS-F ↑PPARγ anti-inflammatory effect	[20]
Multiple myeloma	Inhibits MIP-1α	[26]
Obesity and prediabetes	↑Antioxidant status ↓Lipid and organic peroxides ↓Interferon-γ	[8]
Multiple sclerosis (MS)	Protective role in MS	[33]

ADAMTS-5 (A Disintegrin and Metalloproteinase with Thrombospondin Motifs-5); COX-2 (Cyclooxygenase-2); CXCL-10 (C-X-C Motif Chemokine Ligand 10); GPX (Glutathione Peroxidase); IL-1β (Interleukin-1 beta); IL-17 (Interleukin-17); IL-6 (Interleukin-6); IL-8 (Interleukin-8); INF-γ (Interferon-gamma); LCN2 (Lipocalin-2); MAPK (Mitogen-Activated Protein Kinase); MCS-F (Macrophage Colony-Stimulating Factor); Monocyte chemoattractant protein-1 (MCP-1); MIP-1α (Macrophage Inflammatory Protein-1 alpha); MMP13 (Matrix Metalloproteinase-13); MMP-2 (Matrix Metalloproteinase-2); MS (Multiple Sclerosis); NADPH (Nicotinamide Adenine Dinucleotide Phosphate); NFκβ (Nuclear factor kappa β); NOS-2 (Nitric Oxide Synthase-2); OC (Oleocanthal); PPARγ (Peroxisome Proliferator-Activated Receptor gamma); ROS (Reactive Oxygen Species); SOD (Superoxide Dismutase); TNF-α (Tumor Necrosis Factor-alpha); VEGF/KDR (Vascular Endothelial Growth Factor/Kinase Insert Domain Receptor).

## 5. Oleocanthal and Cancer

**Figure 5:**
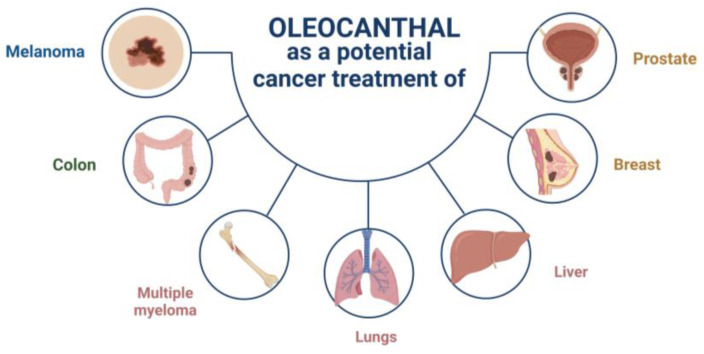
Diagram illustrating various cancers studied and potentially treatable with OC.

In 2011, oleocanthal was first recognized for its remarkable anticancer properties [34]. Elnagar et al. were pioneers in highlighting its potential in inhibiting cell migration, proliferation, and metastasis in breast cancer cells (MCF7 and MDA-MB-231) and prostate cancer cells (PC-3) [34]. After this finding, other investigations in various cancer models were studied (Figure 5 and Table 2). OC demonstrated its promise as a suppressor of breast cancer recurrence. Particularly in the case of triple-negative breast cancer, OC has been found to not only reduce cell growth but also lower the levels of CA 15-3, a recognized marker of recurrence [35].

**Figure 6:**
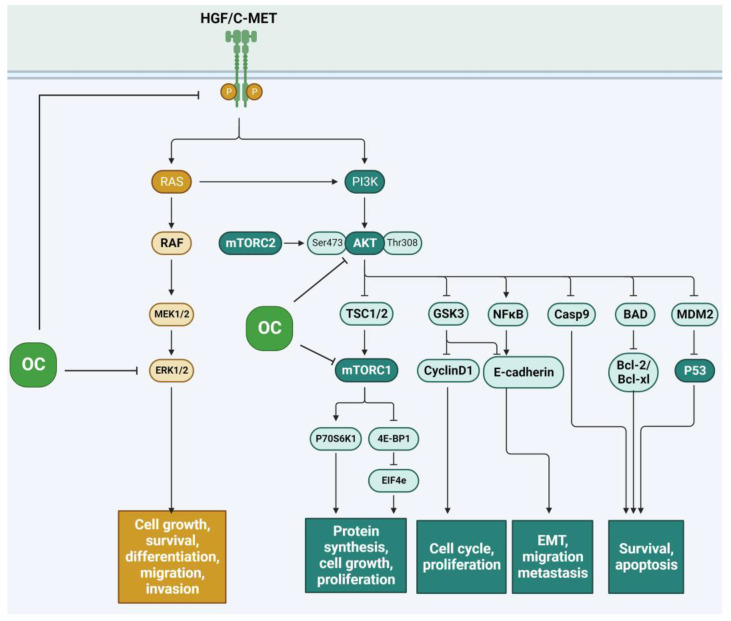
Schematic representation of the inhibition of OC in the HGF/c-Met signaling pathway.

The HGF/c-Met (Hepatocyte Growth Factor/Hepatocyte Growth Factor Receptor) signaling pathway (Figure 6) is frequently reactivated by cancer cells during tumorigenesis, invasive growth, and metastatic progression [36]. The disruption of this signaling pathway leads to alterations in the cytoskeleton of numerous cancer cells and upregulates a variety of functions within these cells, such as cell proliferation, migration, survival, resistance to apoptosis, angiogenesis, invasion, and metastasis [36]. In the human breast, HGF is expressed in mammary stroma, while c-Met is predominantly found in the epithelium. The activation of c-Met by HGF leads to uncontrolled cell growth, potentially giving rise to cancerous states. Prolonged activation of this pathway, on the other hand, is associated with a reduction in E-cadherin expression, a phenomenon linked to tumor invasion. Oleocanthal’s beneficial impact on breast cancer cells and its influence on the c-MET pathway have been extensively documented [37,38]. This effect was also reported to be enhanced in a novel formulation containing xylitol–base liquid–solid (solid dispersion) formulation, showing augmented anti-breast cancer activity [14]. There are various mechanisms through which OC operates to inhibit this pathway. It has been documented that OC can effectively suppress the c-Met signaling receptor in murine models of breast cancer, reducing cell invasion and migration and influencing cell cycle regulation in the G1 phase, ultimately leading to apoptosis [34,39]. It has also been documented that OC serves as a potent inhibitor of this pathway by suppressing the phosphorylation of c-Met, leading to the inhibition of proliferation, migration, and invasion in both breast and prostate cancer [34].

The anti-proliferative effects of OC have been outlined to be regulated by downstream molecules in the c-Met pathway, such as protein kinase B (Akt), mitogen-activated protein kinase (MAPK), signal transducers and transcription activators-3 (STAT-3), and mTOR. Some potential anticancer and neuroprotective effects of OC have been elucidated by their ability to inhibit mTOR. Through a combination of molecular docking and in vitro kinase assays, it has been demonstrated that oleocanthal effectively binds to and inhibits mTOR with a notable IC50 (half maximal inhibitory concentration) value of 708 nM. Oleocanthal has exhibited robust anti-proliferative effects in multiple breast cancer cell lines, accompanied by the downregulation of phosphorylated mTOR in a metastatic breast cancer cell line, specifically MDA-MB-231 [40]. Concerning the STAT-3 pathway, Gu et al. demonstrated that (-)-oleocanthal can inhibit STAT3 signaling in melanoma cancer cells [41]. Similarly, in hepatocellular carcinoma, OC was shown to suppress STAT3 activity [42]. In cutaneous malignant melanoma, although little information is available, Fogli et al. demonstrated that OC was able to inhibit ERK1/2 and AKT phosphorylation and downregulate Bcl-2 expression [43]. In contrast with other polyphenols like oleacein, OC proved to be more efficient and safer as a melanoma treatment [44]. The efficacy of OC in non-melanoma cancer cells was also evaluated; Polini et al. revealed that oleocanthal and oleacein exhibited noteworthy pharmacological effects by proficiently impeding Erk and Akt phosphorylation, concurrently repressing B-Raf expression [45].

It is important to note the lack of absolute curative efficacy treatments for cancer. For this reason, the investigation of treatments for cancers like metastatic castration-resistant prostate cancer (mCRPC), one of the most aggressive prostate cancer (PC) phenotypes, is important. A breakthrough was made when it was found that OC exhibits inhibitory characteristics related to SMYD2 (an enzyme involved in the methylation of protein lysines) and demonstrates positive effects on mCRPC cells [46].

In the context of hepatocellular carcinoma (HCC) and colorectal cancer (CRC) cell lines, OC exhibited remarkable antitumor properties, outperforming the effectiveness of NSAIDs. OC effectively impeded the formation of cell colonies, triggered apoptosis, and incited the generation of intracellular ROS within cancer cells. Through its capacity to induce ROS production, OC provoked damage across various cellular compartments, all while leaving normal cells unaffected. The primary source of OC-induced ROS was identified as NADPH oxidase, and an additional contribution from the mitochondrial respiratory chain complex I, specifically in the form of superoxide anions, was observed [47].

It is worth emphasizing the fact that oleocanthal’s significance also lies in its capacity to inspire the creation of new molecules as potential treatments of cancer. Mohyeldin et al. successfully validated HVS-16, a semi-synthetic secoiridoid analog inspired by oleocanthal, showcasing its c-Met inhibition properties in invasive breast cancer [39].

The effect of oleocanthal on cancer has also been studied as part of a combination anticancer treatment to assess its role as an adjuvant therapy. Specifically, in breast cancer, preclinical in vitro and in vivo studies have been conducted on BT474 cells, in which the combination of OC with tamoxifen (an anti-estrogen) has been characterized by a reduction in the expression of estrogen receptor-alpha (ERα), an effect that could potentially enhance tamoxifen’s effectiveness in inhibiting the growth of breast cancer cells [48].

Another adjuvant treatment that was under investigation involved OC combined with lapatinib, which is a well-known EGFR/HER-2 (Epidermal Growth Factor Receptor/Human Epidermal Growth Factor Receptor 2) inhibitor used for HER-2 amplified breast cancer. This study revealed that the combination of these two substances effectively suppressed the activation of EGFR, HER-2, and c-Met receptors, indicating potential as a treatment for mitigating the invasion and migration of breast cancer cells [49]. It was described that OC promotes calcium (Ca^2+^) entry in MCF7 and MDA-MB-231 cells (breast cancer cells), a process hindered by the suppression of TRPC6 (Transient Receptor Potential Cation Channel Subfamily C Member 6) expression, and also inhibits cell proliferation and migration by modulating calcium ion levels [50].

Other studies have also delved into the synergistic potential of OC when combined with various anticancer treatments. Peri and colleagues conducted a comprehensive investigation into the effects of an enriched extract of extra virgin olive oil, in which OC constituted 55.1% of the total compound, on adenocarcinoma cells. Their research revealed that this extract leads to cell cycle arrest in cancer cells. Intriguingly, the compound’s mode of action appears to involve the stimulation of ROS and the activation of the tumor suppressor protein p53. The study also sheds light on the pivotal role played by Aldo-keto reductases (AKRs) in conferring chemoresistance, while suggesting that the use of OC may complement chemotherapy by enhancing the effectiveness of anticancer drugs [51].

Angiogenesis plays a pivotal role in the development and advancement of cancer by providing tumors with the necessary blood supply to facilitate their growth and spread. Marrero et al. conducted an investigation into the potential of oleocanthal (OC) in the context of angiogenesis treatment. It appears that OC may have a regulatory effect on angiogenesis; however, further research is required to thoroughly elucidate the precise role of this compound [52].

Pastorio et al., in their research, examined the impact of oleocanthal on an acute myeloid leukemia (AML) cell line and an acute lymphoblastic leukemia (ALL) cell line. Their study revealed that OC inhibited the growth of tumor cells and triggered cell death. In AML cells, OC was shown to act through the caspase pathway, while in ALL cells, OC induced cell death independently of the caspase pathway, suggesting the presence of alternative mechanisms of action that remain to be fully elucidated [53].

In their study, Papakonstantinou et al. conducted a comprehensive assessment of the impact of various phenolic compounds derived from EVOO on a diverse panel of cancer cell lines, including MDA-MB 231, SK-BR-3, MCF-7, A2058, SK-MEL-28, AGS, HepG-2, PANC-1, Huh-7, H1299, and Hela. Their findings revealed that oleocanthal exhibited superior antiproliferative and cytotoxic properties in the majority of cell lines, with exceptions observed in two hepatic cancer cell lines, Huh-7 and HepG-2, and the H1437 cell line, where oleuropein and ligstroside aglycones demonstrated greater effectiveness than oleocanthal [54].

**Table 2:** Main findings in studies concerning OC and cancer.

Type of Cancer	Outcome	Ref.
Breast cancer	OC reduces the proliferation of TNBC cells	[12]
	EVOO extract enriched with OC reduces cell proliferation and augments cell death	[13]
	OC-(+)-xylitol solid dispersion formulation enhances antitumoral c-Met activity	[14]
	Inhibits cell migration, proliferation, and migration in the metastatic MCF7 and MDA-MB-231 breast cancer cells	[34]
	Prevents the recurrence of localized and regional breast cancer	[35]
	OC + Tamoxifen: Potentially enhances tamoxifen’s effectiveness in inhibiting the growth of cells ↓Expression of ERα	[48]
	OC + Lapatinib: Inhibits EGFR, HER-2, and c-Met receptors	[49]
	Inhibition of TRPC6, cell proliferation, and cell migration	[50]
Prostate cancer	Inhibits cell migration, proliferation, and migration in PC-3 prostate cancer cells	[34]
	↓PSA levels in mouse model	[46]
Lung cancer	Inhibits c-Met-COX2	[38]
Liver cancer	Inhibits activation of STAT3	[42]
	Inhibits proliferation of human liver cancer cells	[13]
	Inhibits cell colonies, triggers apoptosis, generation of ROS species	[47]
Colon cancer	Generation of ROS species, apoptosis, inhibition of cell colonies	[47]
Colorectal adenocarcinoma	Controls the growth and apoptosis of cancer cells in HT-29 cells through AMPK activation and COX-2 expression	[55]
Gastric adenocarcinoma	Enriched OC oil stimulates reactive oxygen species (ROS) and the activation of the tumor suppressor protein p53	[51]
Neuroblastoma	Inhibition of cell proliferation of neuroblastoma cancer cells	[56]
Melanoma	Inhibition of STAT3 signaling pathway	[41]
	OC induces cytotoxicity against human melanoma ↓ERK1/2 and AKT phosphorylation ↓Bcl-2 expression	[43]
Hematopoietic tumor cells	AML cells: cell death through caspase activation	[53]
	ALL cells: cell death independent of caspase pathway	[53]

AML (Acute Myeloid Leukemia); AKT (Protein Kinase B); ALL (Acute Lymphoblastic Leukemia); AMPK (AMP-Activated Protein Kinase); c-Met (Hepatocyte Growth Factor Receptor); COX-2 (Cyclooxygenase-2); EGFR (Epidermal Growth Factor Receptor); ERα (Estrogen Receptor-alpha); ERK1/2 (Extracellular Signal-Regulated Kinases 1 and 2); EVOO (extra virgin olive oil); HER-2 (Human Epidermal Growth Factor Receptor 2); OC (Oleocanthal); PSA (Prostate-Specific Antigen); p53 (Tumor Protein 53); ROS (Reactive Oxygen Species); STAT3 (Signal Transducer and Activator of Transcription 3); TNBC (Triple-Negative Breast Cancer); TRPC6 (Transient Receptor Potential Cation Channel Subfamily C Member 6).

## 6. Discussion

Extra virgin olive oil (EVOO) has a long history as a key part of the Mediterranean diet, and is known for its great health benefits. Across centuries, *Olea europea* L. have yielded numerous phenolic compounds, captivating the scientific community with their potential. Among these, oleocanthal has recently taken the spotlight, emerging as a promising therapeutic agent against a spectrum of ailments, showcasing its significant pharmacological abilities in diverse disease processes [2,3,4,5]. From quelling inflammation to combating cancers and tackling neurodegenerative disorders, oleocanthal’s medical potential is both intriguing and promising. In this systematic review, we delve into the latest pharmacological evidence supporting oleocanthal’s medical importance, honing our focus on its roles in anti-inflammatory measures and chemotherapy.

In this systematic review, we included all the in vitro and in vivo studies concerning the use of OC as a treatment of inflammation or against several cancer types. The first known characteristic of OC as a potential treatment of inflammation is its inhibition of cyclooxygenase-1 and 2, in a dose-dependent way; for this reason, it has been acknowledged as a naturally occurring NSAID [17,18,19]. Within this context, OC demonstrated the ability to inhibit inflammatory genes, such as IL-6, IL-8, COX-2, NOS-2, MIP-1α, TNF-α, and LCN2, catabolic genes, such as MMP13, and ADAMTS-5 in OA chondrocytes and macrophages [19,26,27]. These data showed the importance of this compound in the treatment of rheumatic diseases, regulating NF-kB and MAPK pathways.

The role of OC in chronic inflammation is important as long as it is considered as an early-stage promoter of carcinogenesis. As we have described in this review, OC showed its anticancer properties through diverse pathways. The HGF/c-Met signaling pathway, frequently reactivated in cancer progression, plays a central role in tumorigenesis, invasive growth, and metastatic advancement. OC emerges as a potent inhibitor of this pathway, effectively suppressing c-Met signaling and impacting cell invasion, migration, and proliferation in both breast and prostate cancer. Additionally, OC’s influence extends to downstream molecules in the c-Met pathway, including Akt, MAPK, STAT-3, and mTOR, where it exhibits anti-proliferative effects. Beyond breast cancer, OC’s efficacy is also explored in melanoma, hepatocellular carcinoma, and colorectal cancer, showcasing its potential as a versatile anticancer agent. The synergy of OC with existing anticancer treatments and its role in adjuvant therapy, especially in breast cancer, underscores its versatility and promise in oncology. Moreover, the impact of OC on angiogenesis, a crucial process in cancer development, is a topic for further investigation. Altogether, this research underscores the diverse and significant potential of oleocanthal in cancer treatment, as well as its capacity to inspire innovative approaches toward combating this complex disease.

Approximately 10% of the overall phenolic composition found in extra virgin olive oil (EVOO) is attributed to OC. Surprisingly, as we described in this review, this seemingly modest percentage appears to play a crucial role in promoting its anticancer properties. Indeed, the consumption of consistent, low-level doses of OC, alongside other phenolic compounds present in EVOO, in alignment with the Mediterranean dietary tradition, has the potential to gradually dampen the body’s inflammatory reactions. This long-term moderation of inflammation may ultimately lead to substantial decreases in the risk of developing chronic inflammatory conditions, including cancer. In contrast with other polyphenols such as oleacein, OC demonstrated superior effectiveness and safety [44].

Furthermore, in the comparison of oleocanthal with other phenolic compounds (including oleuropein aglycone, ligstroside aglycone, oleacein, and oleocanthalic acid), oleocanthal displayed the highest relative antiproliferative and cytotoxic activity across various cancer cell lines [54].

This compelling research finding underscores the potential of OC as a promising candidate in comparison with other EVOO polyphenols. The study of OC holds paramount significance in the quest to find effective treatments for inflammation and cancer. One limitation in these studies is the lack of information on the pharmacokinetics and pharmacodynamics of this phenolic compound. Despite the abundance of in vitro data on OC, a deeper understanding of its potential as a treatment requires additional in vivo studies and clinical trials. Thus, further studies are necessary to fully elucidate the complete role of OC in these contexts.

To sum up, the newly gained insights into the pharmacology of OC and its derivatives present innovative perspectives for cancer and inflammation treatments.

## Data Availability

Data sharing not applicable.
